# l‐Carnitine relieves cachexia‐related skeletal muscle fibrosis by inducing deltex E3 ubiquitin ligase 3L to negatively regulate the Runx2/COL1A1 axis

**DOI:** 10.1002/jcsm.13544

**Published:** 2024-08-02

**Authors:** Zongliang Lu, Li Wang, Zhenyu Huo, Na Li, Ning Tong, Feifei Chong, Jie Liu, Yaowen Zhang, Hongxia Xu

**Affiliations:** ^1^ Department of Clinical Nutrition Daping Hospital, Army Medical University (Third Military Medical University) Chongqing China; ^2^ Chongqing Municipal Health Commission Key Laboratory of Intelligent Clinical Nutrition and Transformation Daping Hospital, Army Medical University (Third Military Medical University) Chongqing China; ^3^ Department of Medical Engineering The 32280 Troops of China People's Liberation Army Leshan China

**Keywords:** cancer cachexia, DTX3L, l‐carnitine, Runx2, skeletal muscle fibrosis

## Abstract

**Background:**

Cancer cachexia‐induced skeletal muscle fibrosis (SMF) impairs muscle regeneration, alters the muscle structure and function, reduces the efficacy of anticancer drugs, diminishes the patient's quality of life and shortens overall survival. RUNX family transcription factor 2 (Runx2), a transcription factor, and collagen type I alpha 1 chain (COL1A1), the principal constituent of SMF, have been linked previously, with Runx2 shown to directly modulate COL1A1 mRNA levels. l‐Carnitine, a marker of cancer cachexia, can alleviate fibrosis in liver and kidney models; however, its role in cancer cachexia‐associated fibrosis and the involvement of Runx2 in the process remain unexplored.

**Methods:**

Female C57 mice (48 weeks old) were inoculated subcutaneously with MC38 cells to establish a cancer cachexia model. A 5 mg/kg dose of l‐carnitine or an equivalent volume of water was administered for 14 days via oral gavage, followed by assessments of muscle function (grip strength) and fibrosis. To elucidate the interplay between the deltex E3 ubiquitin ligase 3L(DTX3L)/Runx2/COL1A1 axis and fibrosis in transforming growth factor beta 1‐stimulated NIH/3T3 cells, a suite of molecular techniques, including quantitative real‐time PCR, western blot analysis, co‐immunoprecipitation, molecular docking, immunofluorescence and Duolink assays, were used. The relevance of the DTX3L/Runx2/COL1A1 axis in the gastrocnemius was also explored in the in vivo model.

**Results:**

l‐Carnitine supplementation reduced cancer cachexia‐induced declines in grip strength (>88.2%, *P* < 0.05) and the collagen fibre area within the gastrocnemius (>57.9%, *P* < 0.05). At the 5 mg/kg dose, l‐carnitine also suppressed COL1A1 and alpha‐smooth muscle actin (α‐SMA) protein expression, which are markers of SMF and myofibroblasts. Analyses of the TRRUST database indicated that Runx2 regulates both COL1A1 and COL1A2. In vitro, l‐carnitine diminished Runx2 protein levels and promoted its ubiquitination. Overexpression of Runx2 abolished the effects of l‐carnitine on COL1A1 and α‐SMA. Co‐immunoprecipitation, molecular docking, immunofluorescence and Duolink assays confirmed an interaction between DTX3L and Runx2, with l‐carnitine enhancing this interaction to promote Runx2 ubiquitination. l‐Carnitine supplementation restored DTX3L levels to those observed under non‐cachectic conditions, both in vitro and in vivo. Knockdown of DTX3L abolished the effects of l‐carnitine on Runx2, COL1A1 and α‐SMA in vitro. The expression of DTX3L was negatively correlated with the levels of Runx2 and COL1A1 in untreated NIH/3T3 cells.

**Conclusions:**

This study revealed a previously unrecognized link between Runx2 and DTX3L in SMF and demonstrated that l‐carnitine exerted a significant therapeutic impact on cancer cachexia‐associated SMF, potentially through the upregulation of DTX3L.

## Introduction

Cancer cachexia is a multifactorial syndrome characterized by an ongoing loss of skeletal muscle mass.^1^ Notably, the reduced muscle mass is also associated with the accumulation of extracellular matrix (ECM), which is synthetized by fibroblasts, resulting in increased fibrous connective tissue and decreased parenchymal cells.^2^ The excessive accumulation of ECM results in skeletal muscle fibrosis (SMF), which leads to structural damage and dysfunction of the muscle, which may become life threatening.^2^ Supporting the importance of SMF, the post‐operative survival rate of pancreatic cancer patients with cachexia who had high SMF was lower than that of patients with low SMF.^3^ The increase in SMF in patients with cancer cachexia is accompanied by an increase in endometrial space,^3^ which is inconsistent in non‐cachexic cancer patients.^S1^ Thus, studying SMF is crucial to better identify cachexia and to monitor its progression.

Dietary counselling, adequate nutritional support and physical exercise are currently the mainstays of treatment for cancer cachexia. A systematic review of the effects of different minerals, vitamins and other supplements on cancer cachexia by the European Palliative Care Research Centre showed that only l‐carnitine (LC) was able to increase the median survival of patients with pancreatic cancer.^4^ Moreover, a decrease in the serum LC level was found to be a biomarker of sarcopenia, which was defined as the loss of skeletal muscle mass, quality and function in cancer patients.^5^ Sarcopenia caused by anticancer drugs may also be reduced by LC, and it has been suggested that sorafenib might inhibit carnitine absorption to induce sarcopenia.^6^ Shang et al. showed that reducing SMF can attenuate sarcopenia in aging mice.^7^ Many studies have demonstrated that LC can reduce the ECM in patients with liver, kidney and heart‐related diseases.^8^, ^9^, ^10^ However, the effects of LC on cancer cachexia‐associated SMF are unclear. LC supplementation may represent a potential strategy for preventing or treating cancer cachexia‐related fibrosis.

Collagen type I alpha 1 chain(COL1A1) is a key structural component of the ECM and the main component of SMF.^11^ Stilhano et al. showed that inhibiting the expression of COL1A1 alleviated skeletal muscle degeneration.^12^ Nintedanib, an EndMT‐inhibiting drug, was shown to improve skeletal muscle function by decreasing the expression of SMF‐related genes, such as COL1A1.^13^ These studies suggest that reducing the expression of COL1A1 may alleviate SMF and thus reduce the muscle wasting and decreased muscle strength caused by cancer cachexia. RUNX family transcription factor 2 (Runx2) is a transcription factor that is considered to be an osteogenic marker and a promising therapeutic target for malignant tumours.^S2^ Ducy et al. showed that Runx2 directly regulated the mRNA level of COL1A1.^14^ Another study indicated that Runx2 expression is upregulated during denervated muscle atrophy.^15^ Recent research showed that Runx2 promotes aortic and aortic valve fibrosis in models of type 2 diabetes and aging.^S3^ ^,^ ^S4^ Transforming growth factor be (TGF‐β1), which is a major factor driving fibrosis, is secreted by cancer cells and promotes the expression and function of Runx2.^16^ Thus, there may be a relationship among TGF‐β1, Runx2 and COL1A1 related to cancer cachexia‐associated SMF.

Deltex E3 ubiquitin ligase 3Leltex‐3‐lik (DTX3L) belongs to the E3 ubiquitinase Deltex family. The previous research on DTX3L has mainly focused on tumours, where it has been shown to be involved in cancer cell survival, proliferation, apoptosis, metastasis and DNA repair.^17^ Recently, several studies have shown that the expression of DTX3L is associated with inflammatory diseases.^S5^ Our previous research showed that LC alleviates the decrease in skeletal muscle mass and inflammation induced by cachexia in mice and activates the AKT (protein kinase B) pathway.^18^ In support of this observation, Hu et al. showed that silencing DTX3L inhibits the AKT pathway.^19^ However, the effects of LC on DTX3L, the role of DTX3L in fibrosis and their relationship with cancer cachexia‐associated SMF are currently unclear.

In this study, we explored whether LC could alleviate SMF and whether the mechanisms involved DTX3L, Runx2 and COL1A1. We believe our findings may provide new insights into cancer cachexia and the potential benefits of LC supplementation.

## Methods

### Mouse model of cachexia

Cachectic mouse experiments were conducted according to a protocol reviewed and approved by the Animal Ethics Committee of the Army Medical University. The study adhered to all relevant regulations regarding animal research. Female BALB/c nude mice (6 weeks old) and C57 mice (44–48 weeks old) were obtained from the breeding animal facilities of the Army Medical University (Chongqing, China). The nude mouse model of cachexia has been described previously.^18^ Briefly, six female nude mice were randomly assigned to control (three) and LC (three) groups based on their body weight, and the cancer cachexia model was established by subcutaneously transplanting 5 × 10^6^ CT26 cells into the left flanks of these mice. Sixteen C57 mice were allowed to acclimate to the animal facility for 1 week and then were randomly assigned to a normal (four), MC38 (six) or MC38 + LC (six) group based on their body weight. A total of 5 × 10^6^ MC38 cells in 0.1 mL of Dulbecco's modified Eagle's medium (DMEM) without foetal bovine serum (FBS) were injected subcutaneously into the right inguinal area of each C57 mouse to induce cancer cachexia in these mice. The treatment (distilled water or 5 mg/kg LC) was administered by oral gavage daily for 14 days. The body weight and tumour size were recorded every 3 days (*Figure* *S1* *A*).

### Cell lines and cell models

CT26, HEK 293 and NIH/3T3 cells were purchased from Guangzhou Cellcook Biotech. The MC38 cells were obtained from Professor Ye Lilin at the Army Medical University. MC38 cells, CT26 cells, HEK 293 cells and NIH/3T3 cells were cultured in a 37°C incubator in an atmosphere of 5% CO_2_. MC38 cells, CT26 cells and HEK 293 cells were maintained in high‐glucose DMEM supplemented with 10% FBS. NIH/3T3 cells were maintained in high‐glucose DMEM supplemented with 10% newborn bovine serum (NBS). The secretion of TGF‐β by cancer cells contributes to the onset of fibrosis in patients with cancer cachexia.^20^ NIH/3T3 cells were inoculated into T25 culture flasks and treated with a final concentration of 10 ng/mL TGF‐β1 (PeproTech, USA) for 24 h (*Figure* *S1* *B*).

### Mouse grip strength assessment

A mouse was placed on the board of a grip strength metre (Ugo Basile, type 47200, Italy), grabbed by its tail and gently pulled back with even force in a straight line. As the mouse was moved backwards, its limbs gripped the grabbing board until the pulling force exceeded its grip strength. The display showed the maximum grip. The average of the three repeated trials was recorded as the muscle strength of the mouse.

### RNA sequencing analysis

Total RNA was extracted from the gastrocnemius muscle of the two groups of nude mice using a commercial kit, and the subsequent sequencing analysis was performed by Majorbio (Shanghai). The differentially expressed genes (DEGs) threshold was a fold change > 2 and a *P* value < 0.05 by DESeq2.

### Histopathology of the gastrocnemius muscle

The gastrocnemius muscles were fixed in 4% paraformaldehyde solution in 0.2 mol/L phosphate‐buffered saline (PBS), followed by washing in PBS and subsequent staining with haematoxylin and eosin (HE) solution. Subsequently, muscle sections were imaged using the ImageJ software.

### Sirius red staining

The gastrocnemius muscles were stained using a Sirius Red Staining Kit (Double Jane; Shanghai, China). The gastrocnemius muscle of the hind leg proximal to the tumour was removed and sectioned in paraffin and then treated with iron haematoxylin, Sirius red, 0.2% acetic acid, anhydrous alcohol, xylene and dibutylphthalate polystyrene xylene. A microscope was used to examine the tissue at ×100 magnification. Image Pro Plus software (Media Cybernetics, USA) was used to measure the intensity of the Sirius red staining.

### Transfection of plasmids and short interfering RNAs

In the GFP‐DTX3L or Flag‐Runx2 overexpression experiments, the empty plasmid was used for mock transfection. For gene knockdown assays, scrambled control short interfering RNA (siRNA) and DTX3L‐siRNA were made by RiboBio Company (Shanghai, China). Plasmid and siRNA transfection were carried out according to the manufacturer's instructions for Lipofectamine 3000 (L3000015, Invitrogen). In brief, 7.5 μL of Lipofectamine 3000 was added to 250 μL of Opti‐MEM medium (31985‐070, Gibco). Then, 5 μg of cDNA or 50 nmol of siRNA, 10 μL of P300 and 250 μL of Opti‐MEM medium were added to a new 1.5‐mL eppendorf (EP) tube. The Lipofectamine 3000 and plasmid mixture was combined at a ratio of 1:1. After 10–15 min at room temperature, 250 μL of the mixture was added into each six‐well plate of cells, which were incubated at 37°C for 24 h. The cDNA and siRNA sequences for the target genes are listed in *Table* *S1*.

### Immunofluorescence assay

Immunofluorescence assays were performed according to the standard protocol, as described previously.^S6^ The HEK 293 cells were grown on coverslips and transfected with GFP‐DTX3L and Flag‐Runx2 plasmids for 48 h. After being washed in PBS, the cells were fixed for 15 min in 4% paraformaldehyde. The cells were permeabilized in 0.1% TritonX‐100, washed and blocked with goat serum for 30 min. After blocking, the cells were incubated with an anti‐Flag rabbit antibody overnight at 4°C, followed by washing with PBS and incubation with a Cy3‐conjugated secondary antibody for 2 h at 37°C. The coverslips were then incubated with 1 μg/mL 4′,6‐diamidino‐2‐phenylindole hydrochloride (DAPI) for 5 min. Finally, the coverslips were washed and mounted using aqueous mounting medium (Santa Cruz, SC‐24941). Isotype‐specific negative controls were included with each staining experiment. The stained cells were mounted and visualized under a Leica Stellaris STED (Leica TCS SP5, Germany). The antibodies used are listed in *Table* *S2*.

### Duolink assay

The processes before blocking were the same as for standard immunofluorescence, as described above. However, for the Duolink assays, the coverslip was blocked with Duolink® Blocking Solution (Sigma Aldrich, DUO9210) for 60 min at 37°C and then incubated with an anti‐Flag rabbit antibody and an anti‐GFP mouse antibody (Beyotime Biotechnology) overnight at 4°C. Next, the cells were incubated with PLUS and MINUS PLA probes for 1 h at 37°C, Duolink® Ligation Buffer for 30 min at 37°C and Amplification Buffer for 100 min at 37°C. The coverslips were washed twice for 10 min per time at room temperature in Wash Buffer, and the slides were mounted with a coverslip using a minimal volume of Duolink® In Situ Mounting Medium with DAPI. The stained cells were mounted and visualized under a Leica Stellaris STED (Leica TCS SP5). The antibodies used are listed in *Table* *S2*.

### Western blot analysis

Western blotting was performed according to the standard protocol, as described previously.^S7^ Protein was extracted from the gastrocnemius of the hind leg distal to the tumour. NIH/3T3 cells exposed to different conditions were lysed in buffer (containing 1000 μL of cell lysate, 20 μL of phosphatase inhibitor, 20 μL of protease inhibitor and 10 μL of phenylmethanesulfonylfluoride or phenylmethylsulfonyl fluoride [PMSF]) and then centrifuged at 12 000 rpm for 15 min at 4°C. The bicinchoninic acid (BCA) method was used to detect the protein concentration. Protein expression was analysed by western blotting. Membranes were first incubated with the primary antibodies, and then these membranes were incubated with the secondary antibodies for 2 h at room temperature. The results were visualized with the ChemiDocTouch™ Imaging System (BIO‐RAD) and analysed using the Image Lab software. The antibodies used are listed in *Table* *S2*.

### RNA sequencing and statistical analyses

After excision, the left gastrocnemius muscles of nude mice were immediately frozen in liquid nitrogen and sent to Shanghai Majorbio Bio‐pharm Technology Co. Ltd. In brief, RNA was extracted using the QIAzol Lysis Reagent (QIAGEN, Dusseldorf, Germany). Total RNA analyses were done using an Agilent 5300 Bioanalyzer (Agilent Technologies, California, USA). Library preparation of 1 μg of RNA was performed using the Illumina NovaSeq Reagent Kit (Illumina, California, USA). cDNA was evaluated, and sequencing was performed using an Illumina NovaSeq 6000 sequencer (Illumina). The raw paired end reads were trimmed and quality controlled by fastp with default parameters. The differential expression analysis was performed using the DESeq2 program.

### Molecular docking and ligand–target interactions

The predicted structures of DTX3L and Runx2 were generated by Alphafold (Google, California, USA). We used AutoDock Vina (The Scripps Research Institute, California, USA) to perform molecular docking. The Docking Web Server (GRAMM) (Center for Computational Biology, University of Kansas, USA) was used for protein–protein docking. Ligand–target interactions were calculated using AutoDock Tools 1.5.7 under default parameters. Finally, the protein–protein interaction figure was generated by PyMOL (Schrödinger, New York, USA).

### Co‐immunoprecipitation assays

A 20‐μL aliquot of magnetic beads (Thermo Fisher Scientific, USA) was washed in cold PBS and then put in 3% bovine serum albumin (BSA) for 1 h at 4°C. Then, the magnetic beads were washed twice again with cold PBS. A total of 500 μL of cell lysates were placed in the magnetic beads and incubated overnight on a shaker at 4°C. The magnetic beads were washed twice with tris‐buffered saline (TBS) after removing the liquid with the magnetic frame. Then 2 × sodium dodecyl sulfate (SDS) was used to resuspend the magnetic beads, which were subsequently boiled in a 100°C water bath for 10 min. Supernatants were collected after removing the magnetic beads from the magnetic frame.

### Quantitative real‐time PCR

Total RNA was extracted from NIH/3T3 cells using the TRIzol reagent and an Ultrapure RNA Kit (Cwbio, Taizhou, China). The cDNA was obtained by reverse transcription. qPCR was performed using SYBR Green (Bimake, Shanghai, China). The study was repeated independently three times for each sample, and all amplification reactions were analysed using the comparative threshold cycle (Ct) method and normalized to the GAPDH mRNA level. All reactions utilized the following thermal cycler conditions: 95°C for 30 s, 39 cycles of a two‐step reaction with denaturation at 95°C for 5 s and annealing at 59°C for 30 s. The primers for the genes are listed in *Table* *S3*.

### Statistical analysis

All values were presented as means ± SEM. Multiple groups were compared using a one‐way analysis of variance (ANOVA) followed by a least significant difference (LSD) test, and comparisons between two groups were conducted using *t*‐tests by the SPSS software (Version 23.0; SPSS Software Inc., Chicago, USA; IBM, USA). *P* values < 0.05 were considered statistically significant.

## Results

### l‐Carnitine relieves cancer cachexia‐related skeletal muscle fibrosis

**Figure 1:**
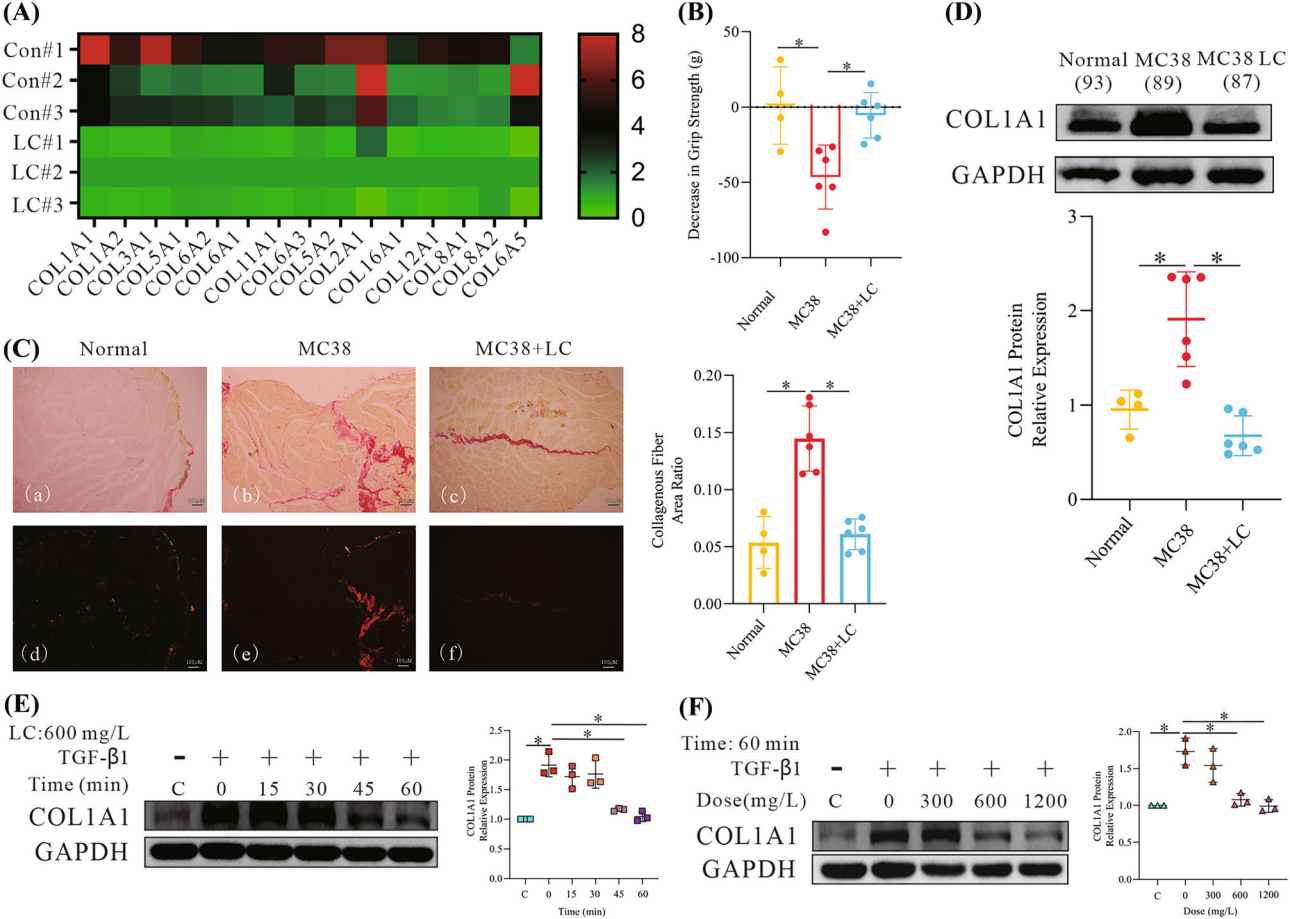
l‐Carnitine (LC) relieves cachexia‐induced skeletal muscle fibrosis (SMF). (A) Changes in the mRNA expression of members of the collagen family in the gastrocnemius muscles of mice with cancer cachexia identified by RNA sequencing in CT26 tumour‐bearing nude mice (*n* = 3, 3). (B) Grip strength assessment in cachectic mice with and without LC treatment (5 mg/kg). The bar graph shows the muscle strength (grip strength) determined by a muscle strength metre (*n* = 4, 6, 6). (C) The collagenous fibre area in cachectic mice with and without LC intervention. The bar graph shows the SMF level in each group (*n* = 4, 6, 6). (D) Western blot (WB) analysis of the collagen type I alpha 1 chain (COL1A1) expression in the gastrocnemius. The bar graph shows the relative COL1A1 protein expression (*n* = 4, 6, 6). (E, F) WB analysis of the COL1A1 expression in NIH/3T3 cells following exposure to 10 ng/mL transforming growth factor beta 1 (TGF‐β1) for 24 h and after subsequent treatment with 600 mg/L LC for different amounts of time or with different concentrations of LC for 60 min. The dot chart shows the relative COL1A1 protein expression. Representative images are shown, and images were assessed using the Image Lab software. The data are shown as the means ± SEM (*n* = 3). A one‐way analysis of variance (B–F) followed by the least significant difference test was used to compare data among groups (*P < 0.05).

Our previous research demonstrated that LC alleviates cancer cachexia‐related muscle wasting.^18^ To further explore the effects of LC, we conducted RNA sequencing (RNAseq) on the gastrocnemius muscles of nude mice. As shown in *Figure* *1* *A*, LC treatment reduced the mRNA level of members of the collagen family. Middle‐aged (44‐ to 48‐week‐old) mice were used for initial studies because the skeletal muscles of middle‐aged mice are more susceptible to loss and have greater difficulty recovering than those of young mice. Liu et al. previously found that the daily administration of 5 mg/kg LC could maintain a normal serum concentration in a mouse model of cachexia.^21^ Therefore, we administered 5 mg/kg LC to each mouse after established tumours had formed. The muscle strength (as indicated by the grip strength) in the MC38 + LC group was similar to that in the normal group, and there were significant differences between these mice and the untreated MC38 group (*Figure* *1* *B*). The total and tumour‐free weights of the mice were not significantly different; however, the results demonstrated that LC supplementation effectively prevented weight loss in the cachectic mice in the MC38 experiment (*Figure* *S1* *C–E*). The gastrocnemius weight and cross‐sectional fibre area demonstrated that the administration of LC prevented muscle atrophy (*Figure* *S1* *F,G*). Moreover, the proportion of gastrocnemius collagenous fibre area in the MC38 group was approximately two times higher than that in the normal and MC38 + LC groups (*Figure* *1* *C*). Further supporting the impact of LC on collagen, the protein expression of COL1A1 in the MC38 + LC group was similar to that in the normal group (*Figures* *1* *D* and *S1* *H*), while the level in the untreated MC38 group was significantly higher.

Based on the physiological concentration of LC in the skeletal muscle of healthy humans,^22^ the cells were treated with 600 mg/L LC. Treatment for 45 min reduced the TGF‐β1‐induced overexpression of COL1A1 (*Figure* *1* *E*). However, a lower concentration of LC did not lead to a significant reduction, and a higher concentration of LC did not lead to any further reduction of COL1A1 (*Figure* *1* *F*). It has been shown that alpha‐smooth muscle actin (α‐SMA), fibronectin and vimentin are markers of the trans‐differentiation of fibroblasts to myofibroblasts.^23^ Treatment of the NIH/3T3 cells with LC for 60 min or 6 h also reduced the TGF‐β1‐induced overexpression of α‐SMA, fibronectin and vimentin (*Figure* *S1* *I*). Notably, treatment of the cells with LC resulted in a concentration‐dependent decrease in the α‐SMA protein level but no significant changes in fibronectin or vimentin (*Figure* *S1* *J*). These results indicate that LC reduces the fibrosis induced by cancer cachexia and maintains skeletal muscle function.

### l‐Carnitine regulates collagen type I alpha chain 1 through RUNX family transcription factor 2 to relieve fibrosis

**Figure 2:**
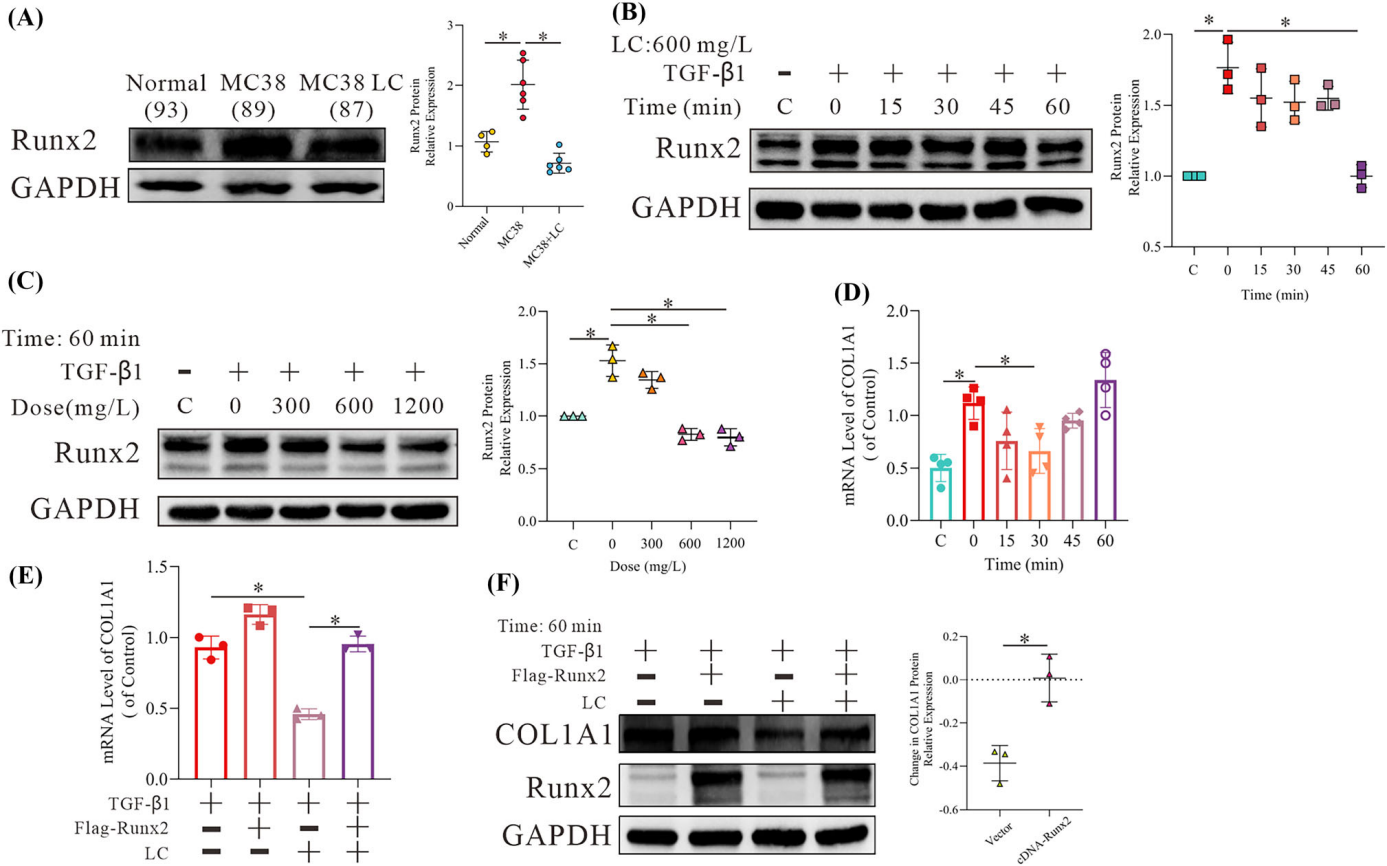
l‐Carnitine (LC) downregulates RUNX family transcription factor 2 (Runx2) to improve fibrosis. (A) Western blot (WB) analysis of Runx2 expression in the gastrocnemius of experimental mice. The bar graph shows the relative Runx2 protein expression (*n* = 4, 6, 6). (B–D) WB and qPCR analysis of the Runx2 and collagen type I alpha 1 chain (COL1A1) expression in NIH/3T3 cells following exposure to 10 ng/mL transforming growth factor beta 1 (TGF‐β1) for 24 h and after subsequent treatment with 600 mg/L LC for different amounts of time or with different concentrations of LC for 60 min. The bar graph shows the relative Runx2 protein and COL1A1 mRNA expression (*n* = 3). (E, F) WB and qPCR analysis of the Runx2 and COL1A1 expression in NIH/3T3 cells transfected with Flag‐Runx2 for 24 h following exposure to 10 ng/mL TGF‐β1 for 24 h and after subsequent treatment with LC for 30 min, 60 min or 6 h. The dot chart shows the relative COL1A1 protein and mRNA levels (*n* = 3). Representative images are shown, and images were analysed using the Image Lab software (A–F). The data are shown as the means ± SEM. A one‐way analysis of variance (A–E) followed by the least significant difference test or *t*‐test (F) was used to compare data among groups (*P < 0.05).

Subsequently, we explored the pathways upstream of the collagen family to determine how LC was exerting its effects. Using the TRRUST database, we found that Runx2 is a key transcription factor that simultaneously promotes the expression of both COL1A1 and COL1A2 (*Figure* *S2* *A*). The study conducted by Raaz et al. demonstrated that Runx2 has the ability to enhance the transcription of both COL1A1 and COL1A2.^S3^ Further analysis of the ‘‐omics’ data revealed a correlation index of 0.9952 between COL1A1 and COL1A2 mRNAs (*Figure* *S2* *B*), and overexpression experiments suggested that Runx2 plays a regulatory role for both COL1A1 and COL1A2 (*Figure* *S2* *C*), supporting that COL1A1 and COL1A2 may be regulated by the same transcription factor. As shown in *Figures* *2* *A,B* and *S2* *D*, LC inhibited the increase in Runx2 protein induced by cancer in both the in vivo mouse model and fibroblasts treated with TGF‐β1. Treatment of the cells with a higher concentration of LC (1200 mg/L) did not have any further effect in vitro, suggesting that the normal physiological level of LC is optimal to reduce fibrosis (*Figure* *2* *C*). As Runx2 promotes the expression of COL1A1 mRNA,^14^ we studied the effects of LC on Runx2‐mediated COL1A1 mRNA expression. As expected, LC decreased the mRNA level of COL1A1 at 30 min, and this effect was abolished when Runx2 was overexpressed (*Figure* *2* *D,E*). Notably, the effects of LC on the COL1A1 protein were abolished by the overexpression of Runx2 (*Figure* *2* *F*). More importantly, the reduction of fibrosis (as indicated by α‐SMA expression) was associated with the effects on Runx2 (*Figure* *S2* *E*).

### l‐Carnitine promotes Runt‐related transcription factor 2 ubiquitination

**Figure 3:**
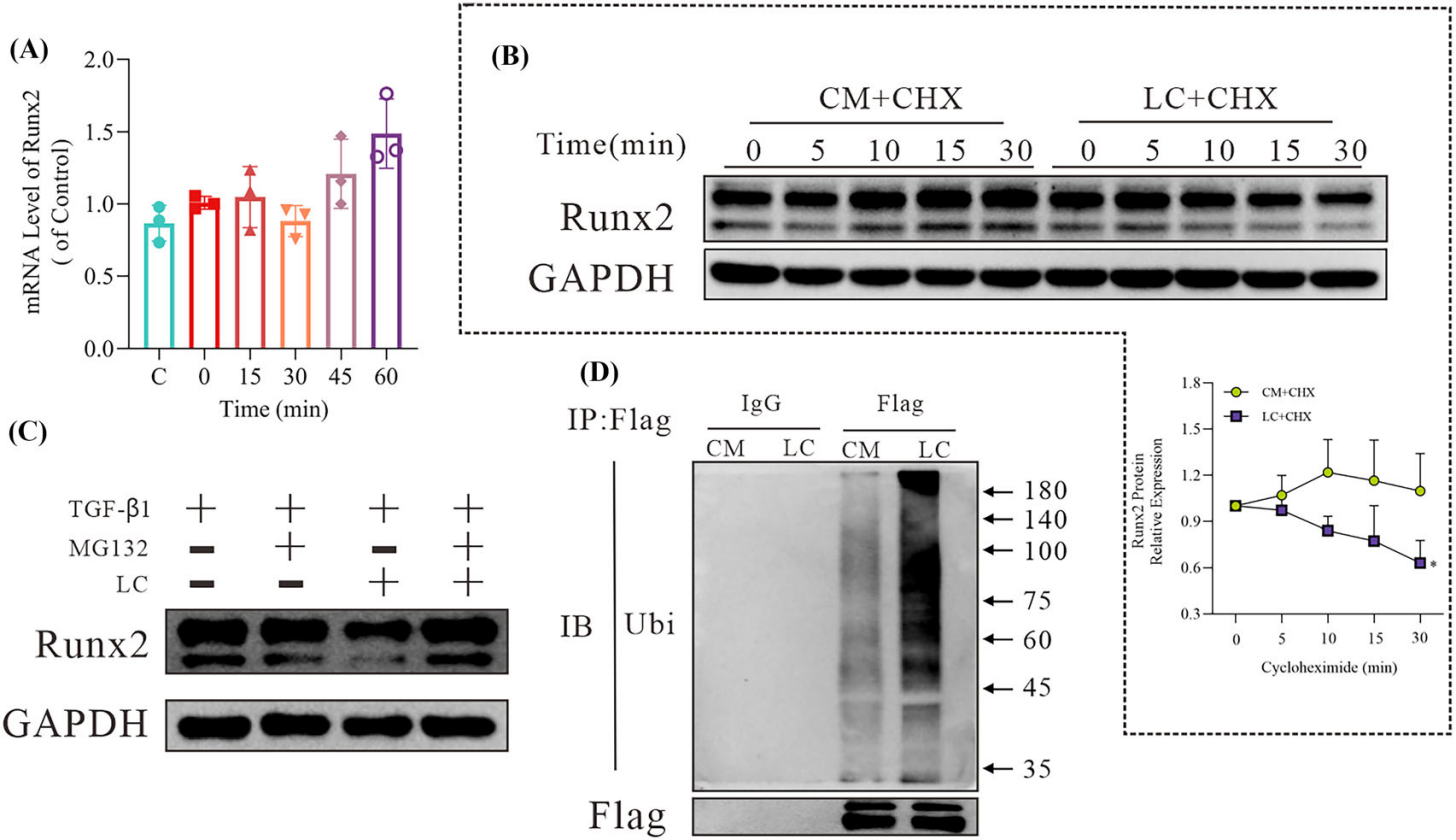
l‐Carnitine (LC) promotes RUNX family transcription factor 2 (Runx2) ubiquitination. (A) qPCR analysis of the Runx2 expression in NIH/3T3 cells following exposure to 10 ng/mL transforming growth factor beta 1 (TGF‐β1) for 24 h and after subsequent treatment with 600 mg/L LC for different amounts of time. The bar graph shows the Runx2 mRNA expression (*n* = 3). (B) Western blot (WB) analysis of the Runx2 expression in NIH/3T3 cells following exposure to 10 ng/mL TGF‐β1 for 24 h in the culture medium (CM) or 600 mg/L LC for 5 min and treatment with 15 mg/L cycloheximide (CHX) for different times. Representative images are shown, and images were analysed using the Image Lab software. A one‐way analysis of variance was used to compare data among the groups (*P < 0.05) (*n* = 3). (C) WB analysis of the Runx2 expression in NIH/3T3 cells following exposure to 10 ng/mL TGF‐β1 for 24 h and treatment with 10 μM/mL MG132 for 12 h, followed by subsequent treatment with 600 mg/L LC for 1 h. (D) WB analysis of ub‐Runx2 expression in NIH/3T3 cells transfected with Flag‐Runx2 for 24 h following exposure to 10 ng/mL TGF‐β1 for 12 h and after treatment with 10 μM/mL MG132 for 12 h and 600 mg/L LC for 15 min based on the enrichment of Runx2 by immunoprecipitation.

We then explored the mechanism by which LC affects Runx2 expression. Interestingly, we observed that LC treatment actually increased the level of Runx2 mRNA (*Figure* *3* *A*). Hence, we examined whether the decreased protein expression of Runx2 was due to increased degradation. Cycloheximide (CHX) is an inhibitor of protein synthesis, and MG132 is a proteasome inhibitor used to prevent protein degradation via the ubiquitin‐proteasome system. As shown in *Figure* *3* *B,C*, LC promoted a decrease in Runx2 protein after treatment with CHX, and this effect was abolished after treatment with MG132, suggesting that LC may promote Runx2 degradation via the ubiquitin‐proteasome system. Further ubiquitination analysis indicated that LC increased the ubiquitination level of Runx2 (*Figure* *3* *D*), supporting that LC increases the ubiquitin‐mediated degradation of Runx2.

### l‐Carnitine regulates RUNX family transcription factor 2 via deltex E3 ubiquitin ligase 3L

**Figure 4:**
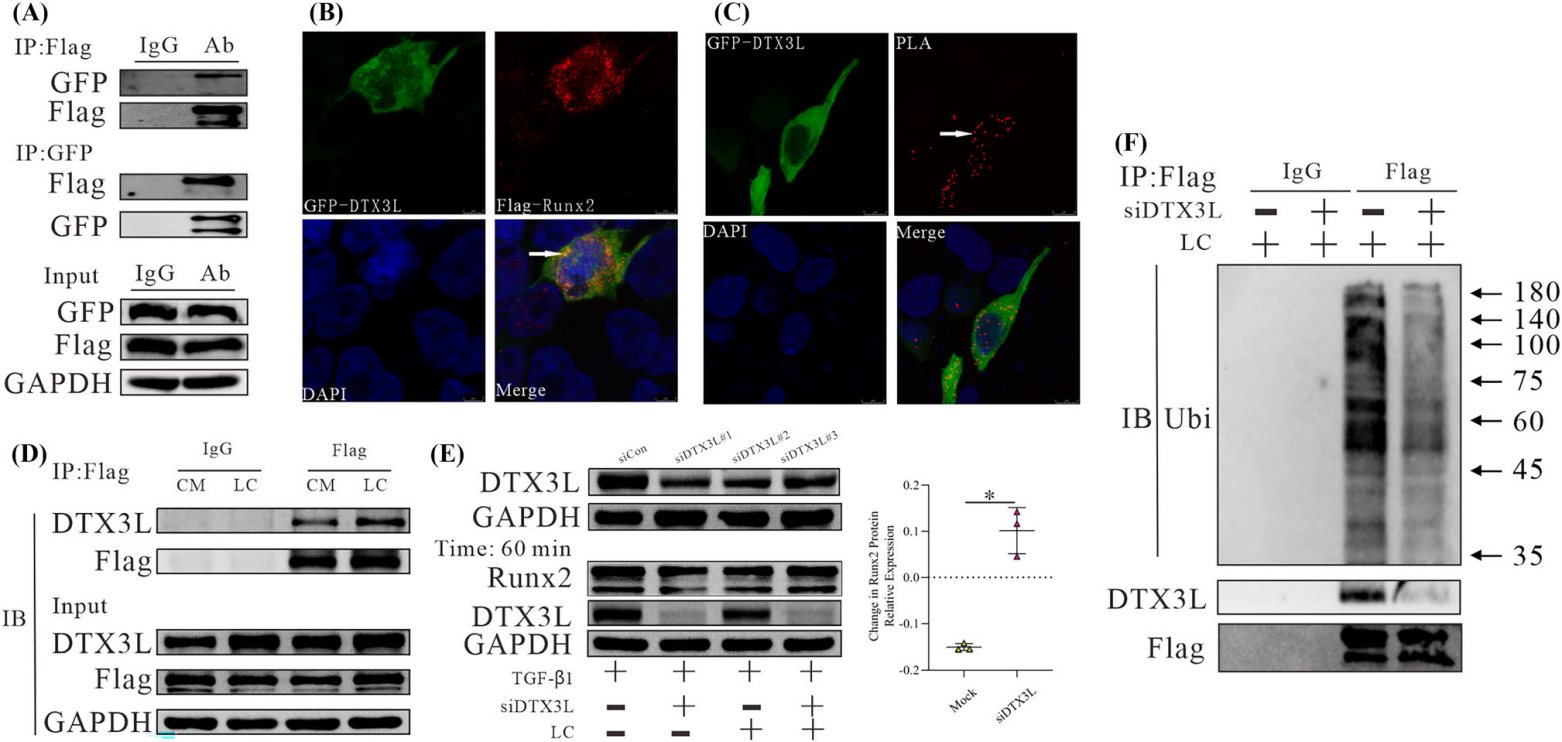
l‐Carnitine (LC) relieves fibrosis via deltex E3 ubiquitin ligase 3L (DTX3L). (A) Western blot (WB) analysis of RUNX family transcription factor 2 (Runx2) and DTX3L in HEK 293 cells after treatment with a Flag‐Runx2 and GFP‐DTX3L plasmid for 24 h after enrichment for Flag or GFP by immunoprecipitation. (B) HEK 293 cells were transfected with Flag‐Runx2 and GFP‐DTX3L for 48 h and then stained with Flag and Cy3 antibodies and counterstained with DAPI to determine whether Runx2 and DTX3L were co‐localized within the cell. Representative images are shown. Arrows indicate the co‐localization of Runx2 and DTX3L. (C) Duolink analysis of the spatial distance between Flag‐Runx2 and GFP‐DTX3L. (D) WB analysis of Runx2 and DTX3L in NIH/3T3 cells after treatment with Flag‐Runx2 for 24 h following exposure to 10 ng/mL transforming growth factor beta 1 (TGF‐β1) for 24 h and 600 mg/L LC for 15 min after enrichment for Flag by immunoprecipitation. (E) WB analysis of the Runx2 expression in NIH/3T3 cells transfected with siRNA‐DTX3L for 24 h following exposure to 10 ng/mL TGF‐β1 for 24 h and after subsequent treatment with LC for 60 min. Representative images are shown, and images were analysed using the Image Lab software. The data are shown as the means ± SEM (*n* = 3). A *t*‐test was used to compare data between groups (*P < 0.05). (F) WB analysis of the ub‐Runx2 expression in NIH/3T3 cells after treatment with Flag‐Runx2 and siRNA‐DTX3L for 24 h following exposure to 10 ng/mL TGF‐β1 for 12 h and treatment with 10 μM/mL MG132 for 12 h and 600 mg/L of LC for 15 min after the enrichment of Flag by immunoprecipitation (*n* = 3).

The ubiquitination of Runx2 requires an E3 ubiquitinase. DTX3L, a known E3 ubiquitinase, was identified to be upregulated based on the RNAseq data (*Figure* *S3* *A*). A PyMOL analysis predicted the presence of multiple binding sites between DTX3L and Runx2, and the score for DTX3L–Runx2 was −651 based on their interaction forces, which is greater than the −400 typically considered a cut‐off for interaction, indicating that there is a very high likelihood of an interaction between the two proteins (*Figure* *S3* *B*). We therefore co‐transfected GFP‐DTX3L and Flag‐Runx2 plasmids into HEK 293 cells and observed that there was indeed a direct interaction between DTX3L and Runx2 based on a co‐immunoprecipitation (CO‐IP) analysis (*Figure* *4* *A*). Immunofluorescence assays showed that DTX3L and Runx2 co‐localized in cells (*Figure* *4* *B*), and Duolink experiments indicated that the space between DTX3L and Runx2 was <40 nm (*Figure* *4* *C*). Notably, treatment with LC increased the direct interaction between DTX3L and Runx2 (*Figure* *4* *D*). After knocking down DTX3L expression with siRNA, LC not only failed to reduce the protein expression of Runx2 but also promoted its increase (*Figure* *4* *E*). Meanwhile, siDTX3L knockdown also reduced the ubiquitination of Flag‐Runx2, supporting that DTX3L is an E3 ligase for Runx2 (*Figure* *4* *F*).

### l‐Carnitine relieves fibrosis through the deltex E3 ubiquitin ligase 3L/RUNX family transcription factor 2 axis

**Figure 5:**
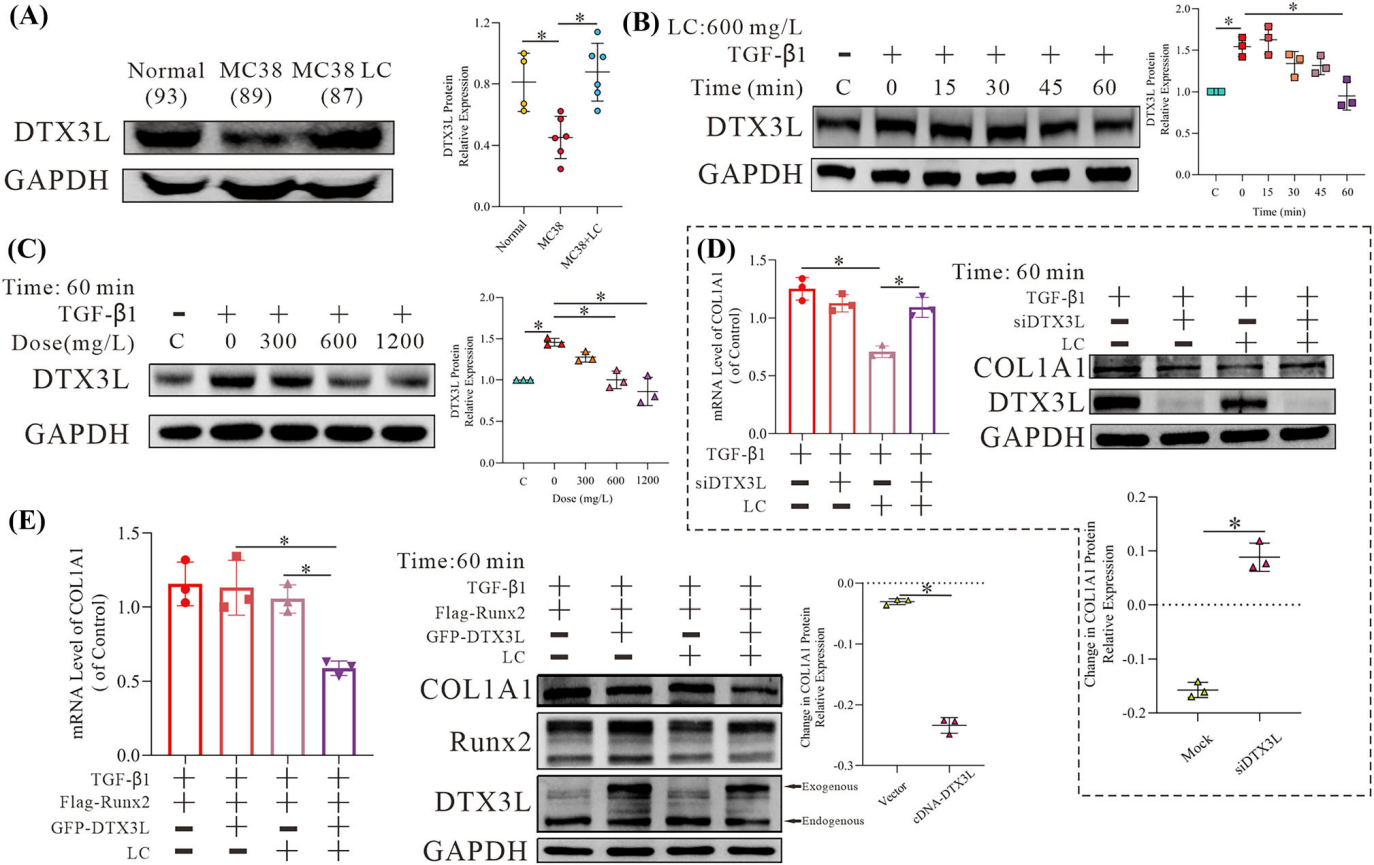
l‐Carnitine (LC) relieves fibrosis through the deltex E3 ubiquitin ligase 3L (DTX3L)/RUNX family transcription factor 2 (Runx2) axis. (A) Western blot (WB) analysis of DTX3L expression in the gastrocnemius. The dot chart shows the relative DTX3L protein expression (*n* = 4, 6, 6). (B, C) WB analysis of the DTX3L expression in NIH/3T3 cells following exposure to 10 ng/mL transforming growth factor beta 1 (TGF‐β1) for 24 h and after subsequent treatment with 600 mg/L LC for different amounts of time or with different concentrations of LC for 60 min. The dot chart shows the relative protein expression of DTX3L (*n* = 3). (D) WB and qPCR analysis of the DTX3L and collagen type I alpha 1 chain (COL1A1) expression in NIH/3T3 cells transfected with siRNA‐DTX3L for 24 h following exposure to 10 ng/mL TGF‐β1 for 24 h and 600 mg/L LC for 30 min, 60 min or 6 h. The bar graph and dot chart show the relative COL1A1 protein and mRNA expression (*n* = 3). (E) WB and qPCR analysis of DTX3L, Runx2 and COL1A1 expression in NIH/3T3 cells transfected with Flag‐Runx2 and either empty vector or GFP‐DTX3L plasmid for 24 h following exposure to 10 ng/mL TGF‐β1 for 24 h and 600 mg/L LC for 30 min, 60 min or 6 h. The bar graph and dot chart show the relative COL1A1 protein and mRNA expression (*n* = 3). Representative images are shown, and images were analysed using the Image Lab software. The data are shown as the means ± SEM (*n* = 3). A one‐way analysis of variance (A–E) followed by the least significant difference test or *t*‐test (D, E) was used to compare data among groups (*P < 0.05).

To determine the role of DTX3L in this model, we checked the expression of DTX3L in mice with cachexia. As shown in *Figures* *5* *A* and *S3* *C*, DTX3L was expressed at an abnormal level in the cachexia model, while LC restored the protein level of DTX3L to the normal (control) level. Similar to Runx2, the DTX3L level was decreased by treatment with 600 mg/L LC for 60 min (*Figure* *5* *B*), and a higher concentration of LC (1200 mg/L) had no further effects on DTX3L expression (*Figure* *5* *C*). As shown in *Figure* *S3* *D*, there was a small increase in the mRNA level of DTX3L, similar to that observed for Runx2. These results suggest that DTX3L plays a role in the response to LC and exhibits changes in expression that correspond to those in Runx2. Moreover, the effects of LC on the protein and mRNA expression levels of COL1A1 were abolished when DTX3L was knocked down with miRNA (*Figure* *5* *D*). This was similar to the effects of LC on α‐SMA and fibronectin, but not vimentin (*Figure* *S3* *E*). Subsequently, both DTX3L and Runx2 were overexpressed in NIH/3T3 cells to confirm whether LC alleviates fibrosis through the DTX3L/Runx2 axis. As shown in *Figure* *5* *E*, the effects of LC on the mRNA and protein expression of COL1A1 were more extensive when DTX3L was overexpressed. Similar results were observed for α‐SMA (*Figure* *S3* *F*). These findings suggest that DTX3L is involved in the mechanism by which LC alleviates fibrosis and imply that DTX3L regulates Runx2.

### Transforming growth factor beta 1 disturbs the deltex E3 ubiquitin ligase 3L/RUNX family transcription factor 2 axis

**Figure 6:**
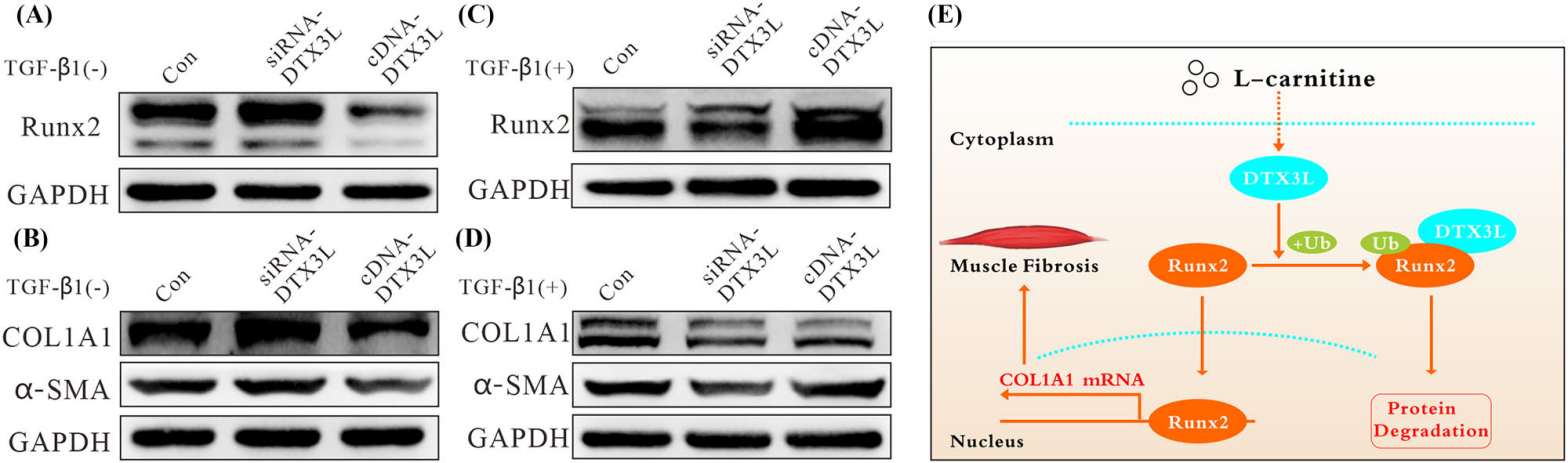
Transforming growth factor beta 1 (TGF‐β1) disturbs the deltex E3 ubiquitin ligase 3L (DTX3L)/RUNX family transcription factor 2 (Runx2) axis. (A, B) Western blot (WB) analysis of the Runx2, collagen type I alpha 1 chain (COL1A1) and alpha‐smooth muscle actin (α‐SMA) expression in NIH/3T3 cells transfected with GFP‐DTX3L or siRNA‐DTX3L for 48 h (*n* = 3). (C, D) WB analysis of the Runx2, COL1A1 and α‐SMA expression in NIH/3T3 cells transfected with GFP‐DTX3L or siRNA‐DTX3L for 24 h, followed by treatment with 10 ng/mL TGF‐β1 for 24 h (*n* = 3). (E) A schematic representation of how l‐carnitine promotes DTX3L to induce Runx2 ubiquitination to relieve cachexia‐related skeletal muscle fibrosis.

Interestingly, we observed a positive correlation between DTX3L and Runx2 (*Figures* *4* *E* and *5* *E*). Therefore, we hypothesized that TGF‐β1 may disturb the DTX3L/Runx2 axis. As shown in *Figure* *6* *A*, the protein expression of Runx2 decreased when DTX3L was overexpressed, and the expression increased after DTX3L was knocked down in cultures without TGF‐β1. Similar results were also observed for the protein expression of COL1A1 and α‐SMA (*Figure* *6* *B*). However, the correlation between the Runx2 and DTX3L proteins shifted from negative to positive when cells were treated with TGF‐β1 (*Figure* *6* *C*). Treatment of cells with TGF‐β1 led to similar effects on COL1A1 and α‐SMA as DTX3L overexpression (*Figure* *6* *D*). These observations suggest that LC partially corrected the DTX3L/Runx2 axis in the model of cancer cachexia‐related fibrosis (*Figure* *6* *E*).

## Discussion

To the best of our knowledge, this is the first study to provide evidence of the effects of LC on cancer cachexia‐related muscle fibrosis and muscle strength. Muscle strength is an important indicator used to evaluate muscle status, and our previous clinical study demonstrated that grip strength is closely related to the mortality of patients with cancer cachexia.^24^ ^,^ ^S8^ ^,^ ^S9^ Clinical studies support that supplemental LC is beneficial to maintain grip strength in haemodialysis patients.^25^ However, supplemental LC did not improve grip strength in healthy elderly people.^26^ These studies show that supplementing LC to provide a normal physiological concentration has a positive impact on grip strength in subjects experiencing a diseased state.

SMF is considered a marker of muscular dystrophy and severe muscle damage^2^ and negatively correlates with grip strength.^24^ ^,^ ^S10^ Previous studies have shown that LC decreases the degree of fibrosis in liver, kidney and cardiac muscle in a variety of disease models.^8^, ^9^ In the present study, we found that LC relieved the SMF caused by cancer. In addition, our previous reports have shown that LC can also improve muscle mass loss in tumour‐bearing mice.^18^ Busquets et al. demonstrated that LC exerts its beneficial effects on the myofibers in subjects with muscle wasting.^27^ ^,^ ^S11^ Further investigations are required to explore the role of LC and its metabolites in different diseases that cause increased SMF expression.

Previous studies have shown that LC may alleviate fibrosis through pathways involving fatty acid desaturase (FADS) 1/2, peroxisome proliferator‐activated receptor gamma (PPARγ) and sirtuin 1 (Sirt1).^8^, ^9^ In this study, we found that LC alleviates fibrosis through the DTX3L/Runx2/COL1A1 axis in our models of cancer cachexia. COL1A1 is a classic marker of SMF.^13^ In Duchenne muscular dystrophy, the expression of the COL1A1 gene can be reduced by a tyrosine kinase inhibitor, nintedanib, and this reduces the degree of SMF.^13^ A previous study in an osteoblast differentiation model also demonstrated that LC treatment affected the mRNA level of Runx2.^28^ We herein reported that DTX3L promoted Runx2 degradation following treatment with LC. Of note, it has previously been suggested that the mechanism underlying the degradation of Runx2 differs based on the cellular circumstances. For example, exposure to tumour necrosis factor‐α (TNF‐α) induces Runx2 degradation through Smurf1 and Smurf2.^29^ Thacker et al. reported that Skp2 regulated the function of Runx2 by promoting its ubiquitination during the differentiation of HEK 293 and MC3T3‐E1 cells into an osteoblast phenotype.^30^ CHIP, as an E3 ubiquitin ligase of Runx2, also played a negative role during the differentiation of MC3T3‐E1 cells into the osteoblast phenotype. Carboxyl terminus of HSC70‐interacting protein (CHIP) not only inhibits the differentiation of MC3T3‐E1 cells into the osteoblast phenotype but also promotes the differentiation of cells into an adipocyte phenotype.^31^ Interestingly, WW domain containing E3 ubiquitin protein ligase 1 (WWP1) induces Runx2 ubiquitination and degradation to inhibit bone formation, while WW domain containing E3 ubiquitin protein ligase 2 (WWP2) promotes Runx2 ubiquitination to induce cell differentiation towards the osteoblast phenotype in HEK 293 and C3H10T1/2 cells.^32^ ^,^ ^S12^

The effects of TGF‐β1 on Runx2 are complex. In mesenchymal precursor cells, TGF‐β1 has been shown to upregulate the expression of Runx2, a key transcription factor in osteoblast differentiation.^33^ TGF‐β1 facilitates the phosphorylation of Runx2. This post‐translational modification plays a critical role in the subsequent activation of matrix metalloproteinase‐13 (MMP‐13), an enzyme implicated in bone resorption and remodelling.^34^ Furthermore, TGF‐β1 enhances the acetylation of Runx2, which is also essential for MMP‐13 expression in osteoblasts.^35^ Recent studies have revealed additional mechanisms through which TGF‐β1 modulates the functions of Runx2. Yu et al. demonstrated that TGF‐β1 can inhibit the degradation of Runx2 in cancellous bone.^36^ Kang et al. identified histone deacetylase 4 (HDAC4) and histone deacetylase 5 (HDAC5) as crucial regulators that suppress Runx2 function during osteoblast differentiation in the presence of TGF‐β.^37^ Moreover, other factors can also influence the acetylation state of Runx2. SIRT6, an NAD^+^‐dependent deacetylase, has been shown to decrease the levels of acetylated Runx2, thereby facilitating the degradation of Runx2.^38^

In contrast, our results show that while DTX3L is also negatively correlated with Runx2, TGF‐β1 changes the relationship between the two proteins. This may be because DTX3L, as an E3 ubiquitin ligase, is an essential carrier for ubiquitin transport and may catalyse different types of ubiquitination, leading to different effects on the substrate. Lo et al. reported that DTX3L promoted ISG15‐mediated ISGylation of lipase (LIPG).^39^ ISGylation is a ubiquitin‐like modification, and its classical role in cells is to maintain the stability of the substrate. Thus, it is possible that the DTX3L‐induced modification of Runx2 may affect the stability of the protein and that the impact of DTX3L on Runx2 is regulated by other factors, such as TGF‐β1. The relationship between MDM2 proto‐oncogene (MDM2) and p53 supports this possibility. The MDM2 protein functions as a ubiquitination enzyme for p53; however, its effects on p53 can vary under different conditions.^40^ ^,^ ^S13^ ^,^ ^S14^ Pan and Chen discovered that MDM2 and MDM4 regulator of p53 (MDMX) collaborate to suppress p53 function in the absence of stress. Following DNA damage, p53 is activated and induces MDM2 expression, subsequently leading to the degradation of MDMX and further activation of p53.^S13^ Yin et al.'s findings suggest that MDM2 enhances the stability and activity of p53 by facilitating its transcriptional upregulation.^S14^ There may be similar conditional regulation of Runx2 by DTX3L.

In summary, our present studies have shown that supplementation to achieve a physiological concentration of LC can improve the decreased muscle strength and increased fibrosis caused by cancer cachexia in C57 mice. This is the first study to find that DTX3L is an E3 ubiquitin ligase for Runx2 in vitro. We also showed that LC regulates Runx2 ubiquitination through DTX3L, leading to a decrease in COL1A1 and alleviating SMF caused by cancer cachexia. Our study provides new targets for the treatment of fibrosis‐related diseases and supports the use of LC supplementation for the treatment of cancer cachexia.

## Conflict of interest statement

The authors declare that they have no known competing financial interests or personal relationships that could have appeared to influence the work reported in this paper.

## Supporting information


**Data S1.** Supporting information.

**Figure S1.** (A) The timing of the *in vivo* experiments in the mouse model of cancer cachexia. (B) An overview of the *in vitro* studies. (C‐F) The body weight, tumor‐free body weight, loss in body weight and gastrocnemius weight in cachectic mice with and without LC intervention. (G) HE analysis of the cross‐sectional area in the gastrocnemius. (H) WB analysis of the COL1A1 expression in the gastrocnemius (*n* = 4,6,6). (I, G) WB analysis of the α‐SMA, vimentin and fibronectin expression in NIH/3T3 cells following exposure to 10 ng/ml TGF‐β1 for 24 h, and after subsequent treatment with 600 mg/L LC for different amounts of time or with different concentrations of LC for 60 min or 6 h. The dot chart shows the relative α‐SMA, vimentin and fibronectin protein expression (*n* = 3). Representative images are shown, and images were assessed using the Image Lab software. The data are shown as the means ±SEM. A one‐way ANOVA followed by the LSD test was used to compare data among groups (* *P* < 0.05).

**Figure S2.** (A) The genes found to be related to the collagen family in the TRRUST database. (B) Correlation between the mRNA expression of COL1A1 and COL1A2 based on RNAseq data. (C) qPCR analysis of COL1A1 and COL1A2 mRNA expression in NIH/3T3 cells transfected with Flag‐Runx2 for 24 h following exposure to 10 ng/ml TGF‐β1 or vehicle for 24 h. (D) WB analysis of Runx2 expression in the gastrocnemius of experimental mice (*n* = 4,6,6). (E) WB analysis of the α‐SMA, vimentin and fibronectin expression in NIH/3T3 cells transfected with Flag‐Runx2 for 24 h following exposure to 10 ng/ml TGF‐β1 for 24 h, and after subsequent treatment with LC for 60 min or 6 h. The dot chart shows the relative α‐SMA, vimentin and fibronectin protein (*n* = 3). Representative images are shown and images were analyzed using the Image Lab software. The data are shown as the means ±SEM. A one‐way ANOVA followed by the LSD test or T‐test was used to compare data among groups (* *P* < 0.05).

**Figure S3.** (A) E3 ubiquitinases among the upregulated genes identified by RNAseq. (B) Molecular docking between DTX3L and Runx2. (C) WB analysis of DTX3L expression in the gastrocnemius (*n* = 4,6,6). (D) qPCR analysis of the DTX3L expression in NIH/3T3 cells following exposure to 10 ng/ml TGF‐β1 for 24 h, and after subsequent treatment with 600 mg/L LC for different amounts of time. The dot chart shows the relative mRNA expression of DTX3L. (E) WB analysis of the α‐SMA, vimentin and fibronectin expression in NIH/3T3 cells transfected with siRNA‐DTX3L for 24 h following exposure to 10 ng/ml TGF‐β1 for 24 h and 600 mg/L LC for 6 h. The bar graph and dot chart show the relative α‐SMA, vimentin and fibronectin protein expression (*n* = 3). (F) WB analysis of the α‐SMA expression in NIH/3T3 cells transfected with Flag‐Runx2 and either empty vector or GFP‐DTX3L plasmid for 24 h following exposure to 10 ng/ml TGF‐β1 for 24 h and 600 mg/L LC for 6 h. The bar graph and dot chart show the relative α‐SMA protein (*n* = 3). Representative images are shown and images were analyzed using the Image Lab software. The data are shown as the means ± SEM. A one‐way ANOVA (F) followed by the LSD test or T‐test (D and E) was used to compare data among groups (* *P* < 0.05).

**Table S1.** Plasmid and siRNA.

**Table S2.** Antibody.

**Table S3.** Primer sequence.

**Data S2.** Supporting information references.
