# Supplementary material for: l‐Carnitine relieves cachexia‐related skeletal muscle fibrosis by inducing deltex E3 ubiquitin ligase 3L to negatively regulate the Runx2/COL1A1 axis
Source: J Cachexia Sarcopenia Muscle. 2024 Aug 2;15(5):1953–64. doi: 10.1002/jcsm.13544 (PMC11446711; doi:10.1002/jcsm.13544)
Supplement: Supplementary file 1 — Data S1. Supporting information. [file JCSM-15-1953-s002.docx]

**LEGENDS**

**Fig. S1.** (A) The timing of the *in vivo* experiments in the mouse model of cancer cachexia. (B) An overview of the *in vitro* studies. (C-F) The body weight, tumor-free body weight, loss in body weight and gastrocnemius weight in cachectic mice with and without LC intervention. (G) HE analysis of the cross-sectional area in the gastrocnemius. (H) WB analysis of the COL1A1 expression in the gastrocnemius (n=4,6,6). (I, G) WB analysis of the α-SMA, vimentin and fibronectin expression in NIH/3T3 cells following exposure to 10 ng/ml TGF-β1 for 24 h, and after subsequent treatment with 600 mg/L LC for different amounts of time or with different concentrations of LC for 60 min or 6 h. The dot chart shows the relative α-SMA, vimentin and fibronectin protein expression (n=3). Representative images are shown, and images were assessed using the Image Lab software. The data are shown as the means ±SEM. A one-way ANOVA followed by the LSD test was used to compare data among groups (* *P*< 0.05).

**Fig. S2.** (A) The genes found to be related to the collagen family in the TRRUST database. (B) Correlation between the mRNA expression of COL1A1 and COL1A2 based on RNAseq data. (C) qPCR analysis of COL1A1 and COL1A2 mRNA expression in NIH/3T3 cells transfected with Flag-Runx2 for 24 h following exposure to 10 ng/ml TGF-β1 or vehicle for 24 h. (D) WB analysis of Runx2 expression in the gastrocnemius of experimental mice (n=4,6,6). (E) WB analysis of the α-SMA, vimentin and fibronectin expression in NIH/3T3 cells transfected with Flag-Runx2 for 24 h following exposure to 10 ng/ml TGF-β1 for 24 h, and after subsequent treatment with LC for 60 min or 6 h. The dot chart shows the relative α-SMA, vimentin and fibronectin protein (n=3). Representative images are shown and images were analyzed using the Image Lab software. The data are shown as the means ±SEM. A one-way ANOVA followed by the LSD test or T-test was used to compare data among groups (* *P*< 0.05).

**Fig. S3.** (A) E3 ubiquitinases among the upregulated genes identified by RNAseq. (B) Molecular docking between DTX3L and Runx2. (C) WB analysis of DTX3L expression in the gastrocnemius (n=4,6,6). (D) qPCR analysis of the DTX3L expression in NIH/3T3 cells following exposure to 10 ng/ml TGF-β1 for 24 h, and after subsequent treatment with 600 mg/L LC for different amounts of time. The dot chart shows the relative mRNA expression of DTX3L. (E) WB analysis of the α-SMA, vimentin and fibronectin expression in NIH/3T3 cells transfected with siRNA-DTX3L for 24 h following exposure to 10 ng/ml TGF-β1 for 24 h and 600 mg/L LC for 6 h. The bar graph and dot chart show the relative α-SMA, vimentin and fibronectin protein expression (n=3). (F) WB analysis of the α-SMA expression in NIH/3T3 cells transfected with Flag-Runx2 and either empty vector or GFP-DTX3L plasmid for 24 h following exposure to 10 ng/ml TGF-β1 for 24 h and 600 mg/L LC for 6 h. The bar graph and dot chart show the relative α-SMA protein (n=3). Representative images are shown and images were analyzed using the Image Lab software. The data are shown as the means ± SEM. A one-way ANOVA (F) followed by the LSD test or T-test (D and E) was used to compare data among groups (* *P* < 0.05).
