# Supplementary material for: l‐Carnitine relieves cachexia‐related skeletal muscle fibrosis by inducing deltex E3 ubiquitin ligase 3L to negatively regulate the Runx2/COL1A1 axis
Source: J Cachexia Sarcopenia Muscle. 2024 Aug 2;15(5):1953–64. doi: 10.1002/jcsm.13544 (PMC11446711; doi:10.1002/jcsm.13544)
Supplement: Supplementary file 8 — Data S2. Supporting information references. [file JCSM-15-1953-s006.docx]

**Supplemental References**

S1.Banduseela V, Ochala J, Lamberg K, Kalimo H, Larsson L. Muscle paralysis and myosin loss in a patient with cancer cachexia. *Acta Myol* 2007;**26**:136-144.

S2.Zhao W, Yang H, Chai J, Xing L. RUNX2 as a promising therapeutic target for malignant tumors. *Cancer Manag Res* 2021;**13**:2539-2548.

S3.Raaz U, Schellinger IN, Chernogubova E, Warnecke C, Kayama Y, Penov K, et al. Transcription Factor Runx2 Promotes Aortic Fibrosis and Stiffness in Type 2 Diabetes Mellitus. *Circ Res* 2015;**117**:513-524.

S4.Chen J, Lin Y, Sun Z. Deficiency in the anti-aging gene Klotho promotes aortic valve fibrosis through AMPKα-mediated activation of RUNX2. *Aging Cell* 2016;**15**:853-860.

S5.Wang L, Sun X, He J, Liu Z. Functions and Molecular Mechanisms of Deltex Family Ubiquitin E3 Ligases in Development and Disease. *Front Cell Dev Biol* 2021;**9**:706997.

S6.Lu Z, Zhou R, Kong Y, Wang J, Xia W, Guo J, et al. S-equol, a Secondary Metabolite of Natural Anticancer Isoflavone Daidzein, Inhibits Prostate Cancer Growth In Vitro and In Vivo, Though Activating the Akt/FOXO3a Pathway. *Curr Cancer Drug Targets* 2016;**16**:455-465.

S7.Lu Z, Song W, Zhang Y, Wu C, Zhu M, Wang H, et al. Combined Anti-Cancer Effects of Platycodin D and Sorafenib on Androgen-Independent and PTEN-Deficient Prostate Cancer. *Front Oncol* 2021;**11**:648985.

S8.Zhuang CL, Zhang FM, Li W, Wang KH, Xu HX, Song CH, et al. Associations of low handgrip strength with cancer mortality: a multicentre observational study. *J Cachexia Sarcopenia Muscle* 2020;**11**:1476-1486.

S9.Yin L, Zhang L, Li N, Guo J, Liu L, Lin X, et al. Comparison of the AWGS and optimal stratification-defined handgrip strength thresholds for predicting survival in patients with lung cancer. *Nutrition* 2021;**90**:111258.

S10.Sosa P, Alcalde-Estévez E, Asenjo-Bueno A, Plaza P, Carrillo-López N, Olmos G, et al. Aging-related hyperphosphatemia impairs myogenic differentiation and enhances fibrosis in skeletal muscle. *J Cachexia Sarcopenia Muscle* 2021;**12**:1266-1279.

S11.Tammo O, Uyanikoglu H, Koyuncu İ. Evaluation of Plasma Free Amino Acid and Carnitine Levels in Patients with Cesarean Scar Pregnancy. *Comb Chem High Throughput Screen* 2021;**24**:1436-1445.

S12.Zhu W, He X, Hua Y, Li Q, Wang J, Gan X. The E3 ubiquitin ligase WWP2 facilitates RUNX2 protein transactivation in a mono-ubiquitination manner during osteogenic differentiation. *J Biol Chem* 2017;**292**:11178-11188.

S13.Pan Y, Chen J. MDM2 promotes ubiquitination and degradation of MDMX. *Mol Cell Biol* 2003;**23**:5113-5121.

S14.Yin Y, Stephen CW, Luciani MG, Fåhraeus R. p53 Stability and activity is regulated by Mdm2-mediated induction of alternative p53 translation products. *Nat Cell Biol* 2002;**4**:462-467.
